# Reassessing After-Hour Arrival Patterns and Outcomes in ST-Elevation Myocardial Infarction

**DOI:** 10.5811/westjem.2015.2.24166

**Published:** 2015-04-02

**Authors:** James Langabeer, Diaa Alqusairi, Jami L. DelliFraine, Ray Fowler, Richard King, Wendy Segrest, Timothy Henry

**Affiliations:** * University of Texas Health Science Center, Houston, Texas; † University of Texas Health Science Center, Division of Management, Policy, and Community Health, Houston, Texas; ‡ Medical University of South Carolina, Department of Healthcare Leadership and Management, Charleston, South Carolina; § UT Southwestern Medical Center, Department of Emergency Medicine, Dallas, Texas; ¶ UT Southwestern Medical Center, Department of Health Care Sciences, Dallas, Texas; || American Heart Association, United States; # Cedars-Sinai Heart Institute, United States

## Abstract

**Introduction:**

Differences in after-hours capability or performance of ST-elevation myocardial infarction (STEMI) centers has the potential to impact outcomes of patients presenting outside of regular hours.

**Methods:**

Using a prospective observational study, we analyzed all 1,247 non-transfer STEMI patients treated in 15 percutaneous coronary intervention (PCI) facilities in Dallas, Texas, during a 24-month period (2010–2012). Controlling for confounding factors through a variety of statistical techniques, we explored differences in door-to-balloon (D2B) and in-hospital mortality for those presenting on weekends vs. weekdays and business vs. after hours.

**Results:**

Patients who arrived at the hospital on weekends had larger D2B times compared to weekdays (75 vs. 65 minutes; KW=48.9; p<0.001). Patients who arrived after-hours had median D2B times >16 minutes longer than those who arrived during business hours and a higher likelihood of mortality (OR 2.23, CI [1.15–4.32], p<0.05).

**Conclusion:**

Weekends and after-hour PCI coverage is still associated with adverse D2B outcomes and in-hospital mortality, even in major urban settings. Disparities remain in after-hour STEMI treatment.

## INTRODUCTION

### Myocardial Infarction and Timeliness of Response

Coronary heart disease is the leading cause of death and healthcare cost in the U.S.^1^ The median lifetime mortality rate is 159.2 for every 100,000 citizens nationally. Among those with heart disease, acute myocardial infarction (MI) is a leading contributor to mortality. The national MI prevalence rate is currently 3.7%, resulting in more than 385,000 annual deaths and costing $108.9 billion dollars in the U.S. each year.^1^–^3^

Time to treatment for patients with ST-elevation myocardial infarction (STEMI) is critical, as it impacts both myocardial salvage and survival.^4^,^5^ Door-to-balloon time (D2B) is a key component of time to treatment and a core quality measure for the Joint Commission.^6^ The American College of Cardiology Foundation/American Heart Association/American College of Cardiology (ACCF/AHA) guidelines recommend a D2B time of no more than 90 minutes from first medical contact to reperfusion.^7^ A recent study reports that from 2005–2010, substantial progress was achieved in national D2B times, with median national D2B times reduced from 96 to 64 minutes.^8^ In particular, when emergency medical services (EMS) activates the cardiac catheterization lab, D2B times can be reduced.^9^

Our analysis seeks to evaluate the impact of arrival times of STEMI patients at PCI-capable hospitals on D2B time and mortality. We specifically compare D2B times between patients who arrived at the hospital during usual business hours and patients who arrived after usual business hours (5pm – 8am). Similarly, we compare D2B times between patients who arrived on weekends and holidays and patients who arrived during working days. Our hypothesis is that patients who arrived after business hours or on weekends will have longer D2B times and higher mortality rates than those who arrived during usual business hours and on weekdays.

### Variation in Quality of Care Across Time of Day and Day of Week

Availability and readiness of healthcare resources, particularly human resources, vary during time of day and day of week. Several studies have investigated the differences in outcome on weekends compared to weekdays and after hours compared to business hours. Some studies have found that there is no difference in outcome based on time of day or week. Miro et al.^10^ and Arabi et al.^11^ found no differences in the effectiveness and quality of care at emergency departments (ED) or intensive care units between weekdays and weekends. Other studies have found that day of admission does have an impact on outcome of care, where mortality was found to be higher on weekends.^12^–^14^ One study with a small sample size of STEMI patients found that patients who arrived at the hospital on weekends or at night had a D2B of >90 minutes while those who arrived during weekday hours had a D2B of <90 minutes.^15^ Two recently published studies found significant relationships between after-hour presentation, D2B and mortality.^16^,^17^ However, many of the studies were not conducted in the U.S., and many studies were of only fair quality, both suggesting more research is needed to examine the relationship between after-hour presentation and mortality. In addition, conflicting research has shown that short-term clinical outcomes for PCI patients were similar, despite longer D2B times in patients receiving after-hour PCI.^18^ Circadian variation has also been shown to impact both frequency of MI onset and infarct size.^19^ Similarly, myocardial infarct size and left ventricular function after STEMI have been shown to have a circadian dependence on the time of day onset of ischemia.^20^ It is quite possible that circadian variation plays a role in patient presentation and treatment times as well.

Our analysis seeks to evaluate the impact of arrival times of STEMI patients at PCI-capable hospitals on D2B time and mortality. We specifically compare D2B times between patients who arrived at the hospital during usual business hours and patients who arrived after usual business hours (5pm – 8am). Similarly, we compare D2B times between patients who arrived on weekends and holidays and patients who arrived during working days. Our hypothesis was that patients who arrived after business hours or on weekends would have longer D2B times and higher mortality rates than those who arrived during usual business hours and on weekdays.

## METHODS AND DATA

### Data Source

Data for this study were collected as part of a project sponsored by the American Heart Association and the W. W. Caruth, Jr. Foundation and the Communities Foundation of Texas to develop a regionally integrated system of care for MI patients in Dallas County, Texas. All of the Dallas County PCI-capable hospitals participated in the collection of the National Cardiovascular Data Registry (NCDR) dataset. NCDR ACTION Registry Get With The Guidelines (GWTG) data were the basis for the hospital component for response time calculations. We collected emergency medical services (EMS) data directly from each EMS agency’s patient care record systems on a quarterly basis. De-identified pre-hospital data were linked to the hospital data using a unique key that joined the data drawn from the EMS incident run number provided by the hospital, which allowed for comparison of time intervals and outcomes. We stored and managed the combined data in a relational database with automated script procedures for suspected matching patient records, importing source files, and validating data within established criteria thresholds. Institutional review board (IRB) approval was obtained and informed consent for limited data set sharing was provided by all participating facilities.

Dallas County is the ninth most populous county in the U.S. Dallas has a very high number of PCI-capable hospitals (15) serving a city population of 1.3 million spanning over 908 square miles, representing a PCI density of 11 hospitals per million capita. This ratio is nearly one-third higher than the median for large urban market.^21^ Data included in this study represent all eligible and complete, non-transfer STEMI patients who presented at the ED and received PCI treatment in Dallas County between October 1, 2010 and September 31, 2012. The age-adjusted mortality rate in Texas caused by AMI is higher than the national average ([52.6 ± 1.0] vs. [41.4 ± 0.2] cases per 100,000, respectively; 95% CI).^22^,^23^

### Statistical Analysis

This study aims to assess the impact of STEMI patients’ arrival times at the PCI-capable hospital on the treatment time as measured by the D2B time and in-hospital all-cause mortality rates. First, we initially grouped patient encounters based on patient characteristics (e.g., age, sex, race), clinical condition upon arrival (cardiac arrest, shock, heart failure – as defined as physician documentation or report of any of the following clinical symptoms of heart failure, or cocaine use at first medical contact), and patient outcomes (length of stay, mortality rate, D2B). Variable definitions can be found in the National Cardiovascular Data Registry (NCDR) dataset. We had four groups (weekend vs. weekday; business hours vs. after hours). The holiday schedule used was obtained from the State of Texas Auditor’s Office.^24^

To compare the D2B times between the different patients groups we initially used the Kruskal–Wallis one-way analysis of variance by ranks, since normality test showed that the dependent variables (D2B and mortality) were not normally distributed. D2B was transformed using the logarithm function to reduce its skewness. For D2B, we used a generalized linear model (GLM) to evaluate the relationships between arrival time variables and treatment time variables, controlling for the patient characteristics, clinical conditions, and arrival mode (EMS arrival vs. walk-in). For mortality, since the data were binary, we used logistic regression with many of the same variables. We used STATA 13 to conduct the statistical analysis.

## RESULTS

For the 1,247 cases included in the analysis: 26% were female, 18% were of Hispanic origin (can be black or white Hispanic, thus some patients checked two ethnicities), 74% were white, and 18% were black. Median age was 59 years. Table 1 shows the demographic characteristics of the patients included in the study, broken down by category. Median D2B for total patients during the study period was 68 minutes, and 77.4% of the patients (n=965) achieved a D2B of less than 90 minutes.

**Figure 1:**
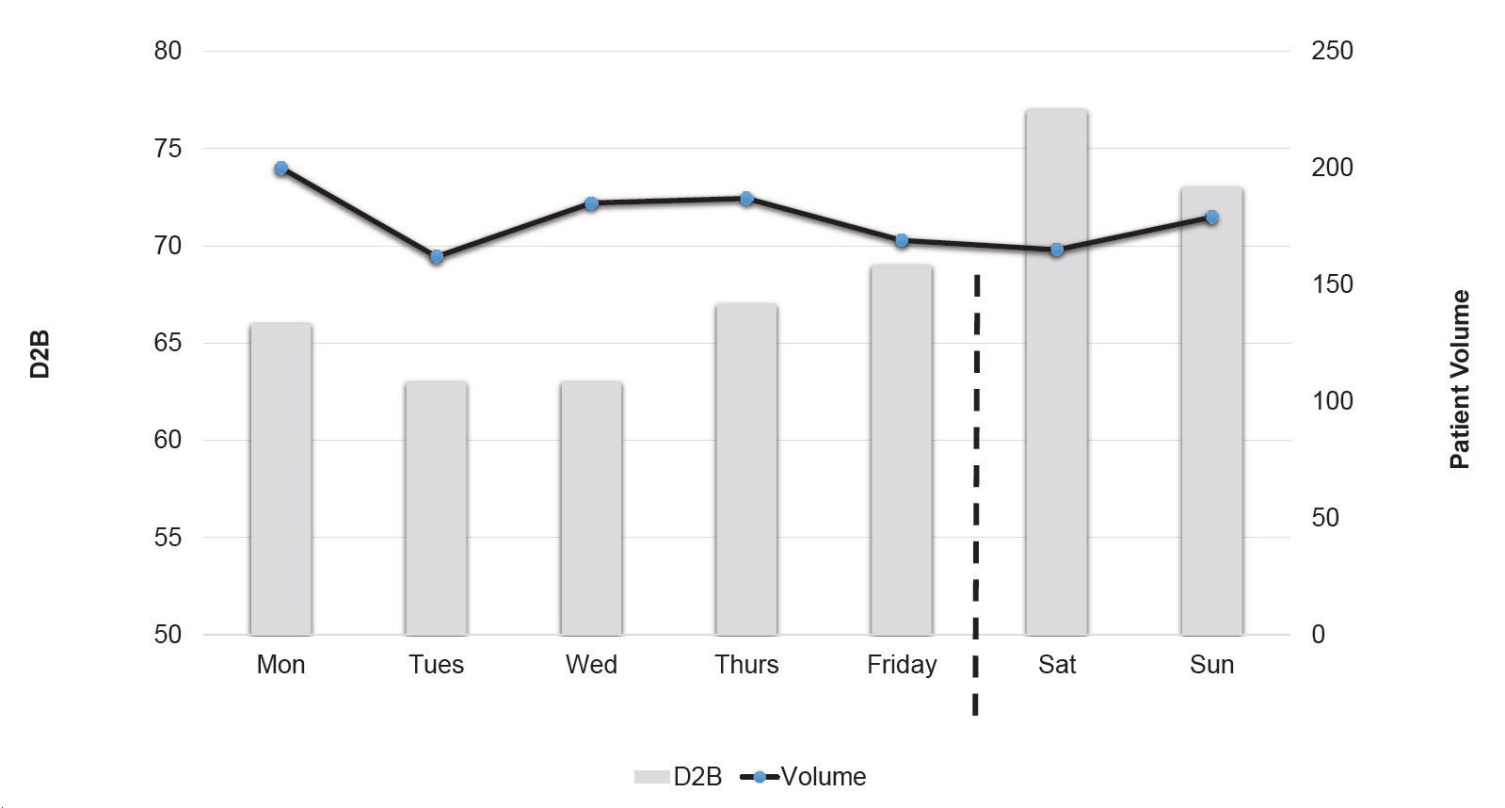
Volumes and median D2B for STEMI patients by day of week. *STEMI,* ST-elevation myocardial infarction; *D2B*, door-to-balloon

Of the 1,247 STEMI patients in the study, 372 (29.8%) arrived at the hospital on weekends or holidays. Figure 1 describes the distribution of patients and median D2B by day of week. The characteristics of patients who arrived at the facility on weekends or holidays were similar to those of patients who arrived on non-holidays weekdays. When looking at the differences in the D2B times, there was a statistically significant difference (KW=48.9, p<0.001) in the D2B time between patients who arrived at the hospital on weekends or holidays compared to those who arrived on non-holiday weekdays. The median D2B for those who arrived at the hospital on weekends or holidays was 75 minutes compared to 65 minutes for those who arrived on non-holiday weekdays (and 75 vs. 59 for after-hours vs. business). D2B times ranged from 12 minutes to 1,152 minutes. Seventy-two percent of patients who arrived on weekends or holidays achieved a D2B time of <90 minutes, compared to 79.5% for those who arrived on weekdays. Figure 1 shows the distribution and median D2B of the number of STEMI patients across the seven days of the week. Comparing the D2B times across the seven days of week yielded statistically significant results (KW=44.5; df=6; p<0.001). D2B times were highest on Saturday and Sunday (77 minutes and 73 minutes respectively) compared to the rest of the days of the week (ranging from 63–69 minutes). Mortality rates were relatively similar in both groups (4.3% vs. 5.2%, and not statistically significant).

To further explore the differences between groups, we applied the generalized linear regression. We used D2B times as the dependent variable, and binary categorical variables for weekend/holiday, business hours, gender, EMS transport, and cardiac catheterization activation by EMS from the field. We controlled for confounding factors by using the presence of cardiac arrest, shock, and heart failure at first medical contact. Patient age was a continuous variable to control for confounding age-related differences.

The regression results confirm that four of these primary variables were statistically significant in the final model: 1) arriving on weekends or holidays (=0.186, p<0.001) and 2) arriving during after-hours (=0.253, p<0.001); 3) transport by EMS vs. patient own vehicle (=−0.150, p<0.001) and 4) cath lab activation by EMS (=−0.302, p<0.001 respectively). The presence of shock and heart failure in patients presentation at first medical contact were both associated with statistically significantly increased D2B time (*=*0.142, p<0.001 and *=*0.106, p<0.001 respectively). All other confounding variables were not associated with statistically significant differences in D2B times.

**Figure 2:**
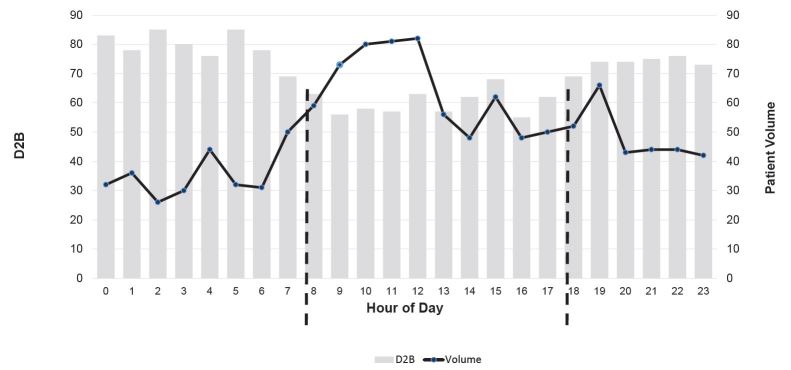
Distribution of STEMI patients by time of day. *STEMI,* ST-elevation myocardial infarction; *D2B*, door-to-balloon

Eighty-three percent of patients who arrived at the hospital during business hours achieved a D2B time of <90 minutes, compared to only 73% for those who arrived after hours. Figure 2 shows the distribution and median D2B times across the time of day. Median D2B times were lower for the period 8am – 5pm compared to the period 5pm – 8am.

Mortality rates were also significantly different in the logistic regression for those patients arriving during after hours (OR 2.23, p<0.05, CI [1.15–4.32]) but not for those who arrived during weekends. The model had a pseudo R^2^=0.36, and a log-likelihood of −158.6, representing a good fit in the model. Confounding control factors for patient age, and presence of shock and cardiac arrest, were significant in the final model as well. Table 2 presents the results of the logistic regression.

## DISCUSSION

We analyzed all 1,247 non-transfer patient cases who were treated for STEMI in Dallas County between October 1, 2010, and September 31, 2012. Our analysis found differences in the D2B times and in-hospital mortality rates based on the day of week and time of day that patients arrived at the hospital. First, patients who arrived on weekends or holidays had a median D2B time 10 minutes longer than those who arrived on non-holiday weekdays (75 and 65 minutes respectively). Also, more patients achieved a D2B timeof <90 minutes on non-holiday weekdays than on weekends or holidays. Second, patients who arrived 8am – 5pm had a median D2B time that was 16 minutes shorter than for those who arrived 5pm – 8am. The 2.23 OR for mortality for patients presenting after hours vs. during business hours is relatively high, compared to the 16-minute difference in median D2B times (75 minutes vs. 59 minutes). This appears to be a disproportionately elevated mortality rate. This also contrasts significantly with the 1.06 OR for mortality for after-hour STEMI presentations in the systematic review and meta-analysis by Sorita et al.,^16^ which demonstrated a similar D2B time delay (14.8 minutes) for after-hour SETMI presentations. However, there were several outlier cases that may explain the mortality discrepancy in this study.

It is interesting to note, as an example, that median D2B at 5am was 85 minutes while 9am was only 56 minutes. As seen in Figure 2, significant treatment time differences exist based on arrival times. Similarly, more patients achieved a D2B of <90 minutes during business hours than after hours (86% and 73% respectively). In-hospital mortality rates were also significantly higher in the after hours than during business hours, with a 1.9 greater odds of dying after hours. Previous studies have shown mixed results regarding the impact of arrival or admission times on the quality of in-hospital care.^16^,^18^ The findings of this analysis, however, are consistent with results from Bell and Redelmeier,^13^ Wichmann et al.,^14^ Mohammed et al.^12^ and Takakuwa et al.^15^

Transport by EMS and field activation of the catheterization lab had a significant relation with D2B, but not with in-hospital mortality. Especially when trying to reduce D2B after hours, the mode of arrival and use of field-based activation of the cardiac catheterization lab appears essential. Results suggest that differences in D2B times and mortality can be influenced by in-hospital operational strategies, such as resource readiness and mobilization protocols. For example, the availability of an attending cardiologist, an emergency medicine physician, nurses, and technicians are well-established factors that affect D2B times.^25^ This availability of key care team members is different across time of day and day of week. Additional investigation of the differences of staffing levels across time will shed more light on why D2B times are different across time.

## LIMITATIONS

This study is one of the larger urban studies of treatment times and arrival patterns. However, it is not without limitations. It is an observational study, not a randomized clinical trial. Although we found a relationship between outcomes and afterhours care, we cannot associate cause and effect. Additionally, although we controlled for multiple factors, we did not examine the hospital or CCL-specific factors that led to this longer treatment time. Although many previous studies have found associations between availability of certain care team members and D2B times, neither previous research nor our study measured the differences of staffing level across time of day or day of week in relation to differences in D2B times. Assessing and quantifying the impact of factors that differ across time on the D2B times will allow for identification of strategies to streamline STEMI treatment processes and reduce variability of D2B times across time of day and day of week.

Finally, these data were collected as part of a program to develop a regionally integrated system of care for patients suffering from acute coronary syndrome. The focus of the program is on reducing mortality and treatment times. This observer effect (or Hawthorne effect) could artificially produce short-term changes in treatment times during the measurement period. In addition, we were not able to measure mortality beyond the in-hospital stay, or control for holidays.

## CONCLUSION

This study found that despite advances in urban STEMI care, disparities remain in after-hours treatment. The arrival time of MI patients at PCI-capable hospitals has an impact on treatment times as measured by D2B. Further investigation of how in-hospital variation affects treatment times will help hospitals streamline care processes across the day and week and reduce associated D2B time variability. Additionally, pre-hospital care was found to significantly improve D2B treatment times, and is especially necessary during after-hours and off-days. This will ultimately help increase the number of patients who receive treatment within the recommended 60–90 minute treatment window.

## Figures and Tables

**Table 1:** Characteristics of STEMI patients by day/time category.

Variable	Weekend/holiday	Weekday	Business hours	Off hours	Total
STEMI patients (n)	372 (30%)	875 (70%)	611 (49%)	636 (51%)	1,247
White	262 (70.4%)	662 (75.7%)	446 (73.6%)	478 (77.8%)	924 (74.1%)
Black	78 (21.0%)	152 (17.4%)	116 (18.5%)	114 (16.2%)	230 (18.4%)
Hispanic	58 (15.6%)	161 (18.4%)	109 (18.5%)	110 (18.3%)	219 (17.6%)
Female	99 (27%)	224 (26%)	155 (25.7%)	168 (25.5%)	323(26%)
Median age	59 (±12.16)	59 (±12.24)	59 (±12.40)	58 (±12.00)	59 (±12.2)
Cardiac arrest at FMC	26 (7.0%)	58 (6.6%)	41 (6.7%)	43 (6.8%)	84 (6.7%)
Heart failure at FMC	46 (12.4%)	101 (11.5%)	67 (10.9%)	80 (12.6%)	147 (11.8%)
Patient in shock at FMC	32 (8.6%)	77 (8.8%)	49 (8.0%)	60 (9.4%)	109 (8.7 %)
EMS transport	194 (52.2%)	432(49.4%)	298 (48.7%)	328 (51.6%)	626 (50.2%)
CCL activated by EMS	59 (15.9%)	124 (14.2%)	94 (15.4%)	89 (14.0%)	183 (14.7 %)
Adjusted length of stay (days)	2.3 (±5.0)	2.2 (±4.3)	2.4 (±5.1)	2.2 (±3.9)	2.2 (±4.5)
D2B (minutes)	75*	65*	59*	75*	68*
D2B<90 minutes	268 (72.0%)	696 (79.5%)	507 (83%)	464 (73%)	965 (77.4%)
In-hospital mortality	4.3%	5.2%	3.6%*	6.3%*	4.97%*

*STEMI,* ST-elevation myocardial infarction; *D2B*, door-to-balloon; *FMC*, first medical contact; *CCL*, cardiac catheterization lab; *EMS*, emergency medical services

* Statistically significant differences, p<0.001.

**Table 2:** In-hospital mortality based on patient arrival on weekends or holidays vs. on non-holiday weekdays.

Mortality	Odds ratio	Standard error	p-value	[95% CI]	
After hours arrival	2.23	0.75	0.017	1.15	4.32
Weekend arrival	0.75	0.28	0.46	0.36	1.59
EMS transport	1.13	0.38	0.70	0.58	2.22
Cath lab activate	0.30	0.18	0.049	0.091	0.99
Age	1.05	0.01	0.000	1.024	1.079
Gender	1.33	0.47	0.41	0.66	2.68
CA_FMC	6.55	2.76	0.000	2.87	14.96
HF_FMC	1.77	0.70	0.15	0.81	3.88
Shock_FMC	15.86	5.89	0.000	7.66	32.86
Cocaine use	1.88	2.20	0.59	0.18	18.70

n=1,247; Psuedo R^2^=0.36, log-likelihood=−158.6

*EMS*, emergency medical services; *FMC*, first medical contact; *CA_FMC*, cardiac arrest at first medical contact; *HF*, heart failure at first medical contact
